# Developing and validating a nomogram for early predicting the need for intestinal resection in pediatric intussusception

**DOI:** 10.3389/fped.2024.1409046

**Published:** 2024-05-07

**Authors:** Yuan-Yang Yu, Jia-Jie Zhang, Ya-Ting Xu, Zheng-Xiu Lin, Shi-Kun Guo, Zhong-Rong Li, Hui-Ya Huang, Xiao-Zhong Huang

**Affiliations:** ^1^ Department of Pediatric Surgery, The Second Affiliated Hospital and Yuying Children’s Hospital of Wenzhou Medical University, Wenzhou, China; ^2^ Department of Intensive Care Unit, The First Affiliated Hospital of Wenzhou Medical University, Wenzhou, China

**Keywords:** intussusception, intestinal necrosis, pediatrics, nomogram, predictor, intestinal resection

## Abstract

**Purpose:**

Develop and validate a nomogram for predicting intestinal resection in pediatric intussusception suspecting intestinal necrosis.

**Patients & methods:**

Children with intussusception were retrospectively enrolled after a failed air-enema reduction in the outpatient setting and divided into two groups: the intestinal resection group and the non-intestinal resection group. The enrolled cases were randomly selected for training and validation sets with a split ratio of 3:1. A nomogram for predicting the risk of intestinal resection was visualized using logistic regression analysis with calibration curve, C-index, and decision curve analysis to evaluate the model.

**Results:**

A total of 547 cases were included in the final analysis, of which 414 had non-intestinal necrosis and 133 had intestinal necrosis and underwent intestinal resection. The training set consisted of 411 patients and the validation cohort included 136 patients. Through forward stepwise regression, four variables (duration of symptoms, C-reaction protein, white blood cells, ascites) were selected for inclusion in the nomogram with a concordance index 0.871 (95% confidence interval: 0.834–0.908).

**Conclusion:**

We developed a nomogram for predicting intestinal resection in children with intussusception suspecting intestinal necrosis after a failed air-enema based on multivariate regression. This nomogram could be directly applied to facilitate predicting intestinal resection in pediatric intussusception suspecting necrosis.

## 1 Introduction

Intussusception, defined as intestinal obstruction caused by the telescoping of a portion of the gut and its mesentery into the adjacent intestinal cavity leading to bowel inflammation and edema (1), is one of the most prevalent causes of acute abdominal diseases in infants and children with peak incidence between 3 and 12 months of age (2). Diagnosing intussusception is challenging depending on the clinical symptoms and signs, which often overlap with multiple other diseases. Furthermore, prompt diagnosis and prediction of intestinal ischemia poses a clinical conundrum for pediatricians and pediatric surgeons due to nonspecific early clinical signs, such as lethargy, diarrhea, or irritability.

Once diagnosis and treatment are delayed, there is a great risk of intestinal ischemia, necrosis, perforation, and peritonitis, even severe dehydration and shock leading to death (3) as the venous congestion and bowel wall edema continue to advance (4, 5). If intestinal necrosis can be predicted and treated earlier with better diagnostic methods, the adverse effects of intussusception can be mitigated, even predicted before complete intestinal necrosis and corresponding treatment can be performed to reduce the incidence of intestinal resection and poor prognosis. Previous studies have focused on the risk factors for intestinal necrosis in intussusception (6, 7), while the complex statistical models limit their utility in clinical decision-making. Therefore, a more accurate and reusable model is urgently needed to predict intestinal resection in intussusception suspecting intestinal necrosis.

Based on multivariate regression analysis, a nomogram integrates multiple predictors and draws them on the same plane according to a certain proportion with calibrated line segments to illustrate the relationship between variables in the prediction model which means more readable and convenient for patients to evaluate (8). Hence, our goal was to develop and validate a nomogram for predicting intestinal resection in pediatric intussusception suspecting intestinal necrosis based on individual preoperative clinical data.

## 2 Methods

### 2.1 Patients

If pediatric patients were highly suspected of having intussusception in outpatient clinic based on ultrasonography and clinical manifestations, a follow-up air enema was performed under fluoroscopy to verify it. Air enema reduction was performed by pediatric surgeons and radiologists up to 3 min with pressure enema from 80 mmHg to 120 mmHg. If first air enema failed, at least two specialized pediatric surgeons would evaluate whether the child was stable, lack of peritonitis, and loosening of the intussusceptum on previous air enema attempt to determine whether to perform air enema again after conservative treatment such as rehydration, anti-inflammatory. If so, after fully informing the family of the risks of re-reduction such as intestinal perforation and obtaining consent, air enema reduction attempt was carried out again. Patients were hospitalized if three air-enema reduction attempts failed or if there was suspicion of intestinal necrosis or perforation. Hospitalized children diagnosed with intestinal intussusception due to unsuccessful air-enema reduction between 1 January 2010 and 31 December 2023 were retrospectively examined. Exclusion criteria were as follows: (1) cases with incomplete medical records; (2) warfarin or heparin use, liver disease, nephrotic syndrome, and/or other diseases potentially affecting blood clotting function.

Emergency surgical reduction was performed after obtaining informed consent in children with failed air-enema reduction. The judgement of intestinal necrosis and the decision to perform intestinal resection were made intraoperatively based on the loss of bowel viability due to non-responsiveness to stimulation, loss of tension and peristalsis, hypoxic discoloration, and the absence of pulsation in the corresponding terminal artery. The final diagnosis of intestinal necrosis depended on the intraoperative findings by surgeons and the postoperative pathological examinations. According to the results of intraoperative and pathology assessments, the enrolled patients were categorized into two groups: the intestinal resection group and the non-intestinal resection group. The patients were randomly selected for training and validation sets in a 3:1 ratio.

The study was approved by the Ethics Committees of The Second Affiliated Hospital and Yuying Children's Hospital of Wenzhou Medical University. Because of the nature of retrospective study, the informed consent was waived.

### 2.2 Variables

The medical records of enrolled patients were collected, including demographic distribution [age, gender, duration of symptoms (hours)], clinical characteristics [red currant jelly stool, fever, abdominal pain, diarrhea, vomitus, intermittent crying, pathological lead points (PLPs), classification of intussusception based on localization (ileo-colic, ileo-cecal, compound/complex, multiple, and small intestinal)], laboratory parameters [C-reaction protein (CRP), white blood cells (WBCs), red blood cells (RBCs), hemoglobin (HB), hematocrit (HCT), mean corpuscular volume (MCV), red cell distribution width-CV (RDW-CV), platelets (PLTs), mean platelet volume (MPV), platelet distribution width (PDW), thrombocytocrit (PCT), neutrophil counts, lymphocyte counts, basophil counts, monocyte counts, eosinophil counts, D-dimer, prothrombin time (PT), prothrombin time-INR (PT-INR), activated partial thromboplastin time (APTT), thrombin time (TT), plasma fibrinogen], ultrasonographic features (ascites, intestinal dilation, enlarged lymph nodes), pneumatic reduction records, surgical findings, and pathology reports. The duration of symptoms was defined as the time from presence of symptoms to the time of hospitalization. Fever was defined as temperature above 37.5°C, as recorded by ear at presentation. Ileo-colic intussusception referred to distal ileum prolapsed into the adjacent colon. Ileo-cecal intussusception referred to distal ileum prolapsed into the adjacent ileocecal junction. Compound/complex intussusception referred to more than one proximal intestinal segment prolapsed into the adjacent one. Multiple intussusception referred to more than one intussusceptions in different intestinal segments. Small intestinal intussusception referred to small intestinal segment prolapsed into the adjacent distal small intestine.

### 2.3 Statistical analysis

The data in this study was analyzed by R4.3.1 software and the Statistical Package for Social Science (SPSS) version 22.0 (IBM, Armonk, New York). There was no missing data in the final analysis. Univariate and multivariate analysis were conducted to estimate the correlation between the predictors and the main binary classification results. Discrete variables were expressed as counts (percentage), and continuous variables as means ± standard deviation (SD) or median with interquartile range (IQR) depending on normality. The normally distributed continuous variables were compared by Student's *t*-test, non-normally distributed variables as Mann–Whitney *U*-test, and the qualitative data as Pearson's Chi-square or Fisher's exact tests. Then, based on clinic and univariable analysis, the significant variables were selected for inclusion in multivariable logistic regression prediction model. Odds ratios (ORs) and 95% confidence interval (CI) were calculated via forward stepwise regression conducted through the maximum likelihood estimation and the model was visualized using a nomogram with the training set, showcasing its calibration and discrimination. The validation set was used to validate the newly developed nomogram. Receiver operating characteristic (ROC) curve evaluated the prediction ability of the model. Area under curve (AUC) >0.5 indicated well prediction value of the model. The concordance-index (C-index) quantifies the level of concordance between predicted and observed probabilities. The calibration curve demonstrated consistency between the prediction and observation. Decision curve analysis (DCA) assessed the clinical risk and benefit. The Hosmer-Lemeshow test was conducted to determine the goodness of fit of the model. A *p*-value <0.05 was considered statistically significant.

## 3 Results

### 3.1 Clinical characteristics

From 1 January 2010 to 31 December 2023, 8,127 pediatric patients were performed air-enema reduction due to intussusception in outpatient clinic, 7,374 cases underwent successful air enema reduction left hospital after a certain period of observation in outpatient clinic if no discomfort occurred during this period, only 753 cases were admitted into the Department of Pediatric Surgery due to air-enema reduction fails or suspicion of intestinal necrosis or perforation. Based on consistent inclusion and exclusion criteria, out of the 753 hospitalized pediatric patients, we excluded 69 cases with incomplete medical records and 137 hospitalized observation cases, who were successfully treated with outpatient air enema. Therefore, the successful rate of air-enema reduction was 92.4% in our hospital. Ultimately, 547 pediatric patients were enrolled in the retrospective analysis, and 133 cases were intestinal necrosis confirmed by pathology. The cases were randomly selected for training and validation sets with a split ratio of 3:1. 411 cases were assigned to the training set, while 136 cases were assigned to the validation set. The training set was used to develop a nomogram model, and the validation set was used to validate the model.

There were no statistical differences between the training set and validation set (*p* > 0.05) in intestinal resection, demographic distribution, and clinical characteristics, as shown in Table 1. Vomitus was the most prevalent presenting symptom (*n* = 488, 89.2%), followed by red currant jelly stool (*n* = 407, 74.4%), intermittent crying (*n* = 400, 73.1%), fever (*n* = 238, 43.5%), abdominal pain (*n* = 106, 19.4%), diarrhea (*n* = 81, 14.8%), and PLPs (*n* = 84, 15.4%) in all 547 enrolled cases. Five types of intussusception based on anatomic localization were observed during surgery: ileo-colic (*n* = 263, 48.1%), ileo-cecal (*n* = 117, 21.4%), compound/complex (*n* = 95, 17.4%), multiple (*n* = 2, 0.4%), small intestinal (*n* = 70, 12.8%). Table 2 summarized the laboratory investigation of the two sets and Table 3 exhibited the sonographic investigation in pediatric intussusception patients of the two sets. 44.4% (243/547) of patients were found ascites free in abdominal cavity or trapped between intestinal loops under ultrasound. 20.8% of (114/547) patients had bowel dilation and enlarged lymph nodes were found in only 7.7% (42/547) of cases. Comparison results between intestinal resection and non-intestinal resection were shown in Tables 4, 5. The duration of symptoms before presentation was significantly longer in children with enterectomy (49.17 ± 29.65 vs. 20.77 ± 14.87, *p* < 0.001). There was no significant difference in abdominal pain, intermittent crying, PLPs, and classifications of intussusception between intestinal resection and non-intestinal resection. Compared with children in non-intestinal resection group, the incidences of fever, bloody stool, diarrhea, and vomitus were higher in children who underwent enterectomy due to suspected intestinal necrosis.

**Table 1:** Demographic and clinical features of the pediatric patients with intussusception with air-enema failure in the training set and validation set.

Variables	Training set *n* = 411	Validation set *n* = 136	*P*-value
Intestinal resection, *n* (%)	104 (25.3)	29 (21.3)	0.4106
Demographics			
Age (month), mean ± SD	15.81 ± 23.91	17.6 ± 26.98	0.6251
Gender (female/male), (ratio)	140/271 (1.0:1.9)	42/94 (1.0:2.3)	0.5636
Duration of symptoms (h), mean ± SD	27.96 ± 23.22	30.98 ± 39.73	0.5813
History, *n* (%)			
Red currant jelly stool	303 (73.7)	104 (76.5)	0.6008
Fever	177 (43.1)	61 (44.9)	0.7912
Abdominal pain	82 (20.0)	24 (17.6)	0.6425
Diarrhea	54 (13.1)	27 (19.9)	0.0764
Vomitus	366 (89.1)	122 (89.7)	0.9570
Intermittent crying	301 (73.2)	99 (72.8)	1.0000
PLPs	66 (16.1)	18 (13.2)	0.5129
Classification of intussusception, *n* (%)			
Ileo-colic	195 (47.4)	68 (50.0)	0.6760
Ileo-cecal	86 (20.9)	31 (22.9)	0.7337
Compound/complex	71 (17.3)	24 (17.6)	1.0000
Multiple	2 (0.5)	0 (0.0)	1.0000
Small intestinal	57 (13.9)	13 (9.6)	0.2476

PLPs, Pathological lead points.

**Table 2:** Hematological investigation of the pediatric patients with intussusception with failed air-enema in the training set and validation set.

Variables	Training set *n* = 411	Validation set *n* = 136	*P*-value
CRP, mg/L	14.97 ± 22.46	15.57 ± 24.89	0.6199
WBCs, 10^9^/L	12.67 ± 5.08	12.78 ± 4.78	0.5656
RBCs, 10^12^/L	4.4 ± 0.69	4.4 ± 0.6	0.4496
HB, g/L	115.39 ± 13.21	115.96 ± 12.77	0.6235
HCT	0.34 ± 0.04	0.35 ± 0.04	0.4531
MCV, fL	78.69 ± 5.32	78.6 ± 6.94	0.5977
RDW-CV, %	13.3 ± 1.84	13.5 ± 2.19	0.6386
PLTs, 10^9^/L	393.68 ± 125.87	400.55 ± 130.40	0.6388
MPV, fL	8.03 ± 1.04	8.04 ± 1.00	0.8976
PDW, %	14.55 ± 2.55	14.44 ± 2.70	0.8824
PCT	0.31 ± 0.10	0.32 ± 0.11	0.6682
Neutrophil counts, 10^9^/L	8.73 ± 4.3	8.7 ± 4.29	0.9996
Lymphocyte counts, 10^9^/L	2.92 ± 1.44	3.08 ± 1.59	0.3721
Monocyte counts, 10^9^/L	0.83 ± 0.59	0.82 ± 0.55	0.8190
Basophil counts, 10^9^/L	0.04 ± 0.05	0.05 ± 0.11	0.8577
Eosinophil counts, 10^9^/L	0.05 ± 0.08	0.07 ± 0.11	0.4866
D-dimer, μg/mL	2.05 ± 2.73	1.75 ± 1.9	0.1208
PT, s	13.85 ± 1.16	13.82 ± 1.03	0.8699
PT-INR	1.07 ± 0.12	1.08 ± 0.11	0.3422
APTT, s	38.58 ± 5.48	38.36 ± 6.02	0.4809
TT, s	15.41 ± 2.87	15.7 ± 3.69	0.1065
Fibrinogen, g/L	3.28 ± 0.83	3.21 ± 0.84	0.3386

Data were presented as mean ± SD. CRP, C-reaction protein; WBCs, white blood cells; RBCs, red blood cells; HB, hemoglobin; HCT, hematocrit; MCV, mean corpuscular volume; RDW-CV, red cell distribution width-CV; PLTs, platelets; MPV, mean platelet volume; PDW, platelet distribution width; PCT, thrombocytocrit; PT, prothrombin time; PT-INR, prothrombin time-INR; APTT, activated partial thromboplastin time; TT, thrombin time.

**Table 3:** Sonographic investigation of the pediatric patients with intussusception with air-enema failure in the training set and validation set.

Variables	Training set *n* = 411, *n* (%)	Validation set *n* = 136, *n* (%)	*P*-value
Ascites			0.9868
Yes	182 (44.3)	61 (44.9)	
No	229 (55.7)	75 (55.1)	
Intestinal dilation			0.8372
Yes	87 (21.2)	27 (19.9)	
No	324 (78.8)	109 (80.1)	
Enlarged lymph nodes			0.4446
Yes	29 (7.1)	13 (9.6)	
No	382 (92.9)	123 (90.4)	

**Table 4:** Demographic and clinical features of intestinal resection group and non-intestinal resection group in the training set.

Variables	Intestinal resection *n* = 104	Non-intestinal resection *n* = 307	*P*-value
Demographics			
Age (month), mean ± SD	15.31 ± 27.08	15.98 ± 22.79	0.806
Gender (female/male), (ratio)	43/61 (1.0:1.4)	97/210 (1.0:2.1)	0.070
Duration of symptoms (h), mean ± SD	49.17 ± 29.65	20.77 ± 14.87	<0.001
History, *n* (%)			
Red currant jelly stool	85 (81.7)	218 (71.0)	0.032
Fever	58 (55.8)	119 (38.8)	0.002
Abdominal pain	18 (17.3)	64 (20.8)	0.435
Diarrhea	21 (20.2)	33 (10.7)	0.014
Vomitus	99 (95.2)	267 (87.0)	0.020
Intermittent crying	71 (68.3)	230 (74.9)	0.186
PLPs	21 (20.2)	45 (14.7)	0.184
Classification of intussusception, *n* (%)			
Ileo-colic	35 (33.7)	160 (52.1)	<0.001
Ileo-cecal	21 (20.2)	65 (21.2)	0.832
Compound/complex	25 (24)	46 (15)	0.035
Multiple	1 (1.0)	1 (0.3)	0.443
Small intestinal	22 (21.2)	35 (11.4)	0.013

PLPs, pathological lead points.

**Table 5:** Hematological and sonographic investigation of intestinal resection group and non-intestinal resection group in the training set.

Variables	Intestinal resection *n* = 104	Non-intestinal resection *n* = 307	*P*-value
CRP	28.15 ± 33.67	10.51 ± 14.67	<0.001
WBCs	13.89 ± 6.31	12.26 ± 4.53	0.016
RBCs	4.32 ± 1.08	4.43 ± 0.49	0.168
HB	113.58 ± 15.04	116.01 ± 12.50	0.105
HCT	0.33 ± 0.04	0.35 ± 0.04	0.009
MCV	79.39 ± 5.00	78.45 ± 5.42	0.119
RDW-CV	13.14 ± 2.16	13.35 ± 1.72	0.316
PLTs	426.39 ± 145.52	382.60 ± 116.68	0.006
MPV	7.95 ± 0.99	8.06 ± 1.05	0.364
PDW	14.31 ± 2.81	14.63 ± 2.45	0.297
PCT	0.34 ± 0.12	0.31 ± 0.09	0.011
Neutrophil counts	9.38 ± 5.02	8.51 ± 4.02	0.113
Lymphocyte counts	3.18 ± 1.61	2.83 ± 1.37	0.032
Monocyte counts	0.98 ± 0.69	0.78 ± 0.54	0.006
Basophil counts	0.043 ± 0.064	0.035 ± 0.040	0.214
Eosinophil counts	0.047 ± 0.067	0.057 ± 0.081	0.264
D-dimer	3.602 ± 4.038	1.529 ± 1.837	<0.001
PT	14.212 ± 1.528	13.732 ± 0.984	0.003
PT-INR	1.114 ± 0.157	1.059 ± 0.104	0.001
APTT	38.005 ± 5.238	38.775 ± 5.557	0.216
TT	15.118 ± 1.168	15.514 ± 3.248	0.225
Fibrinogen	3.243 ± 0.925	3.299 ± 0.793	0.587
Ultrasound, *n* (%)			
Ascites	78 (75)	104 (33.88)	<0.001
Intestinal dilation	36 (34.62)	51 (16.61)	<0.001
Enlarged lymph nodes	7 (6.73)	22 (7.17)	0.881

Continuous variables were described as mean ± SD. CRP, C-reaction protein; WBCs, white blood cells; RBCs, red blood cells; HB, hemoglobin; HCT, hematocrit; MCV, mean corpuscular volume; RDW-CV, red cell distribution width-CV; PLTs, platelets; MPV, mean platelet volume; PDW, platelet distribution width; PCT, thrombocytocrit; PT, prothrombin time; PT-INR, prothrombin time-INR; APTT, activated partial thromboplastin time; TT, thrombin time.

### 3.2 Development of the nomogram

**Figure 1:**
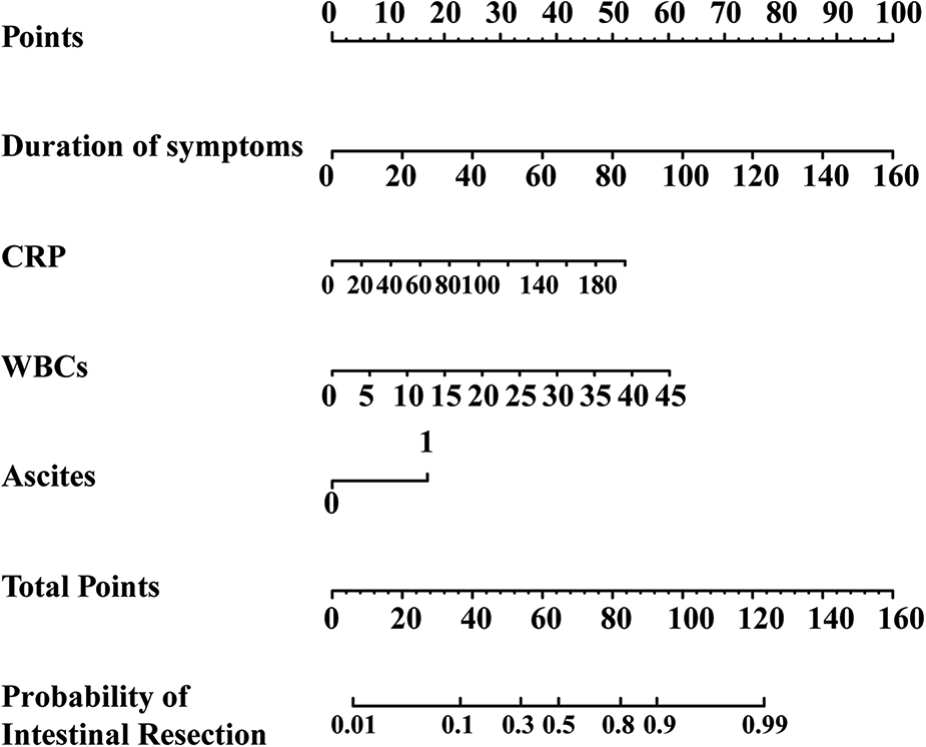
The nomogram for predictors of intestinal resection in children with intussusception after failed air-enema reduction.

Univariate analysis was conducted on the training set and 17 variables were the significant factors (*p* < 0.05), as shown in Tables 6, 7. Based on clinical and statistical evaluation, we included the 17 variables into multivariable analysis and four variables including duration of symptoms (OR = 1.050; 95% CI, 1.035–1.066; *p* < 0.001), CRP (OR = 1.021; 95% CI, 1.007–1.035; *p* = 0.004), WBCs (OR = 1.110; 95% CI, 1.048–1.176; *p* < 0.001), and ascites (OR = 3.781; 95% CI, 2.079–6.874; *p* < 0.001) were screened to develop the nomogram, as illustrated in Figure 1.

**Table 6:** Univariate and multivariate analysis on the demographics and clinical characteristics of the predictors of intestinal resection in pediatric intussusception in the training set.

Variables	Univariate analysis		Multivariate analysis	
	OR [95% CI]	*p*-value	OR [95% CI]	*p*-value
Demographics				
Age	0.999 [0.989–1.008]	0.806		
Gender (male)	0.655 [0.414–1.036]	0.071		
Duration of symptoms	1.060 [1.046–1.074]	<0.001	1.050 [1.035–1.066]	<0.001
History				
Red currant jelly stool	1.826 [1.048–3.182]	0.033		
Fever	1.992 [1.270–3.124]	0.003		
Abdominal pain	0.795 [0.446–1.416]	0.436		
Diarrhea	2.101 [1.153–3.827]	0.015		
Vomitus	2.966 [1.138–7.731]	0.026		
Intermittent crying	0.720 [0.443–1.172]	0.187		
PLPs	1.208 [0.647–2.256]	0.552		
Classification of intussusception, *n* (%)				
Ileo-colic	0.656 [0.358–1.202]	0.173		
Ileo-cecal	0.601 [0.300–1.202]	0.150		
Compound/complex	1.630 [0.821–3.236]	0.162		
Multiple	1.561 [0.089–27.413]	0.761		
Small intestinal	1.886 [0.919–3.871]	0.084		

PLPs, pathological lead points.

**Table 7:** Univariate and multivariate analysis on the hematological and sonographic investigation of the predictors of intestinal resection in pediatric intussusception in the training set.

Variables	Univariate analysis		Multivariate analysis	
	OR [95% CI]	*p*-value	OR [95% CI]	*p*-value
CRP	1.037 [1.024–1.050]	<0.001	1.021 [1.007–1.035]	0.004
WBCs	1.063 [1.018–1.110]	0.006	1.110 [1.048–1.176]	0.002
RBCs	0.737 [0.485–1.120]	0.153		
HB	0.986 [0.970–1.003]	0.106		
HCT	0.000 [0.000–0.160]	0.010		
MCV	1.035 [0.991–1.082]	0.119		
RDW-CV	0.993 [0.814,1.069]	0.318		
PLTs	1.003 [1.001–1.004]	0.002		
MPV	0.903 [0.726–1.125]	0.364		
PDW	0.953 [0.876–1.037]	0.263		
PCT	16.276 [1.841–143.900]	0.012		
Neutrophil counts	1.047 [0.995–1.101]	0.078		
Lymphocyte counts	1.176 [1.013–1.367]	0.034		
Monocyte counts	1.742 [1.211–2.506]	0.003		
Basophil counts	27.686 [0.380–2,017.874]	0.129		
Eosinophil counts	0.159 [0.006–4.090]	0.267		
D-dimer	1.128 [1.005–1.267]	0.040		
PT	1.396 [1.148–1.697]	0.001		
PT-INR	33.734 [4.959–229.485]	<0.001		
APTT	0.974 [0.935–1.015]	0.216		
TT	0.873 [0.726–1.050]	0.150		
Fibrinogen	0.922 [0.702–1.210]	0.556		
Ultrasound				
Ascites	5.856 [3.542–9.682]	<0.001	3.781 [2.079–6.874]	<0.001
Intestinal dilation	2.657 [1.606–4.397]	<0.001		
Enlarged lymph nodes	0.935 [0.387–2.257]	0.881		

CRP, C-reaction protein; WBCs, white blood cells; RBCs, red blood cells; HB, hemoglobin; HCT, hematocrit; MCV, mean corpuscular volume; RDW-CV, red cell distribution width-CV; PLTs, platelets; MPV, mean platelet volume; PDW, platelet distribution width; PCT, thrombocytocrit; PT, prothrombin time; PT-INR, prothrombin time-INR; APTT, activated partial thromboplastin time; TT, thrombin time.

### 3.3 Validation of the nomogram

**Figure 2:**
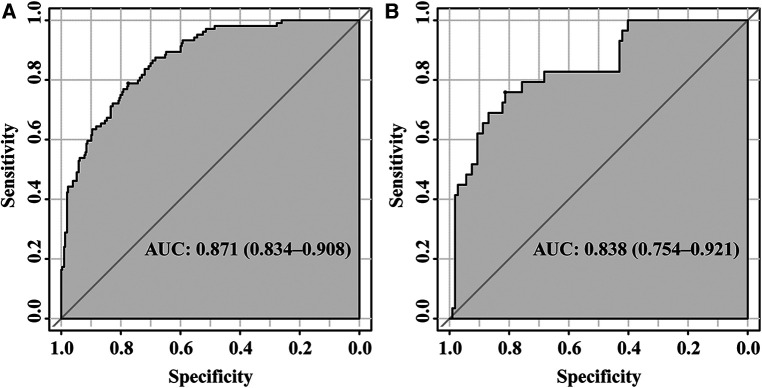
ROC of the established model in the training set (**A**) and validation set (**B**) ROC, receiver operation characteristic; AUC, area under the curve.

**Figure 3:**
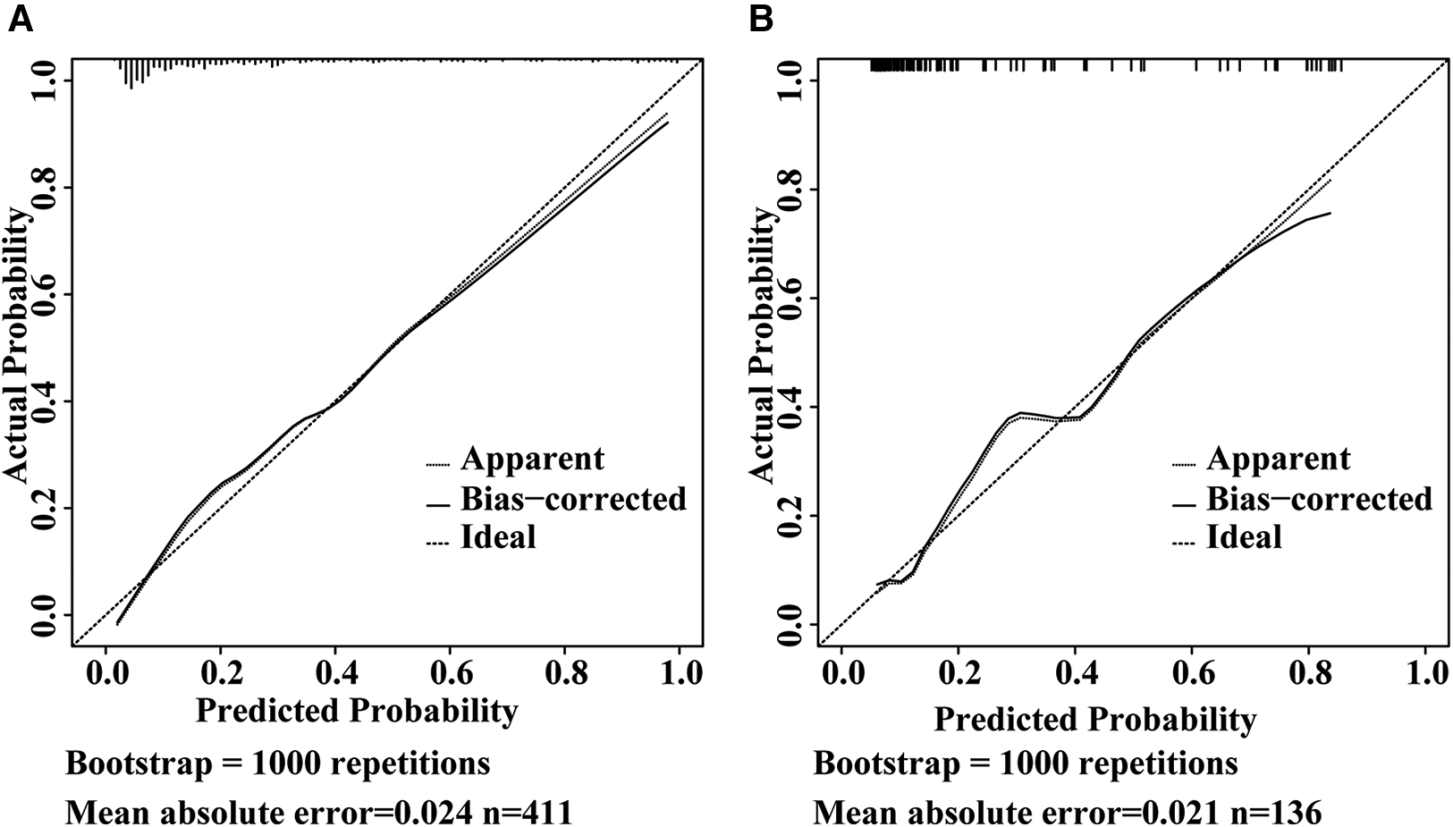
Calibration curve of the model in the training set (**A**) and validation set (**B**) the Y-axis represents the actual intestinal resection rate and the X-axis represents the predicted risk of intestinal resection in pediatric intussusception. The dashed line represents a perfect prediction by an ideal model. The solid line (bias-corrected) represents the bootstrap-corrected performance of our nomogram, and the dotted line (apparent) represents the apparent accuracy of the nomogram.

**Figure 4:**
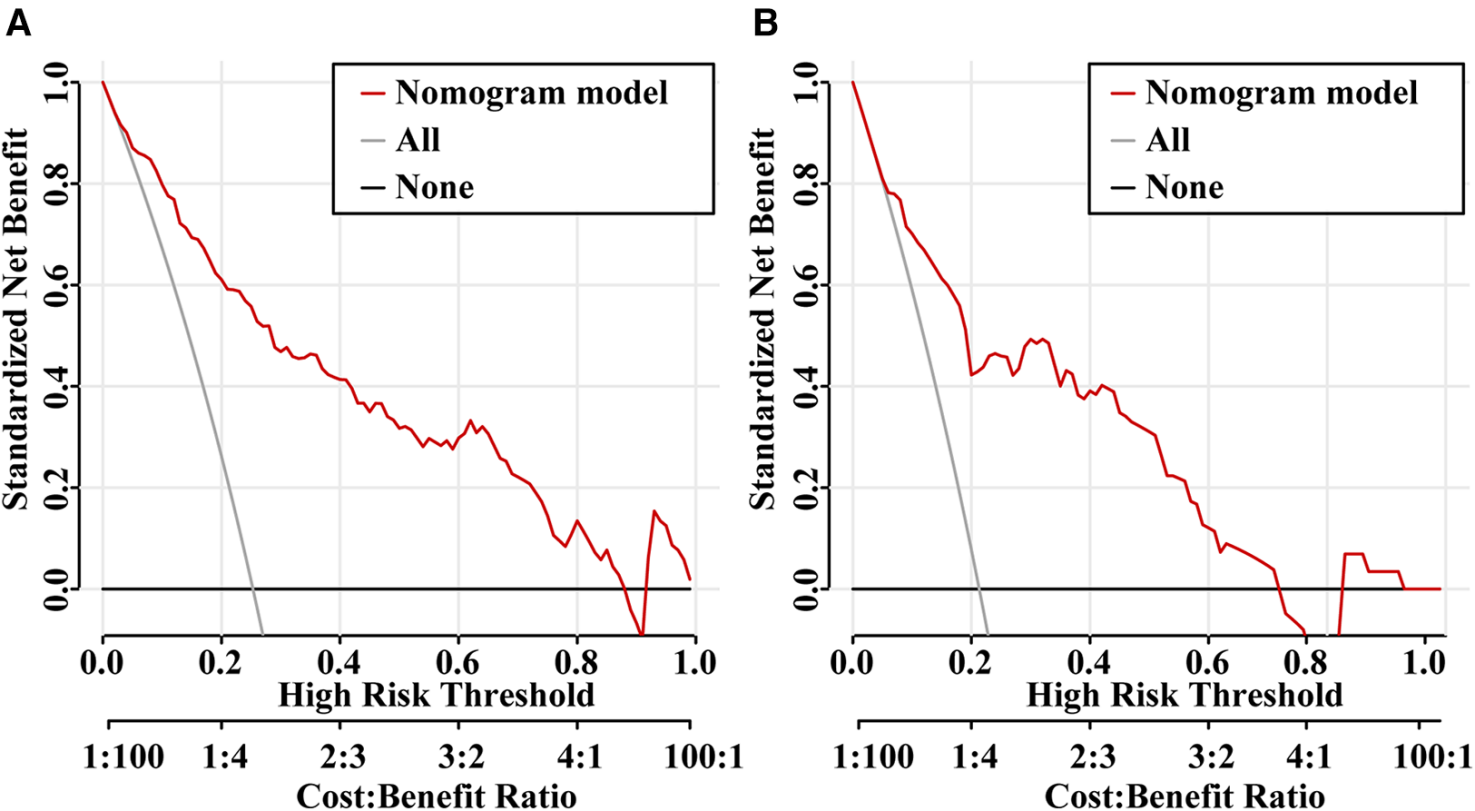
Decision curve analysis (DCA) for the predictive model in the training set (**A**) and validation set (**B**) the Y-axis is the standardized net benefit and the X-axis denotes the threshold of intestinal resection in pediatric intussusception. The red line represents the predictive model. The application of this predictive model would add net benefit compared with either the treat-all or the treat-none strategies.

The ROC curves were plotted, and the AUC of 0.871 (95% CI, 0.834–0.908) in the training set and 0.838 (95% CI, 0.754–0.921) in the validation set were obtained, indicating that the model possessed high predictive value (Figure 2). The C-index of the prediction nomogram in the training set was 0.871 (95% CI, 0.834–0.908), and the Hosmer-Lemeshow test resulted in *p* = 0.716, indicating the good reliability of the forecast and goodness of fit. The calibration curve demonstrated the forecast was in good agreement with the actual situation (Figure 3). In order to assess the model's clinical applicability, we performed decision curve analysis on the nomogram. The DCA showed that the model yielded a net benefit over the majority of threshold probabilities (Figure 4).

## 4 Discussion

Intussusception is one of the most common abdominal emergency in children and due to its anatomical state, occlusion advances through the mechanisms of obstruction and strangulation (9). As the occlusion level increases, intestinal circulation is impaired, leading to ischemia and necrosis (10). Predicting and diagnosing intestinal necrosis in intussusception can be laborious. Scholars are devoting more attention to building a model for facilitating prediction. Compared with screening for predicting risk factors for intestinal necrosis in pediatric intussusception as shown in previous studies (5, 6, 11, 12), a nomogram integrates more variables and replaces complex statistical models with a simple and straightforward graph (13). Furthermore, in recent years, some researchers have expressed grave concern about the predictive ability of nomograms for intussusception and have stated their potential predictive utility (14, 15). To our knowledge, there are few studies focusing on developing nomograms for intestinal resection due to suspected intestinal necrosis in intussusception. Hence, an effective nomogram based on preoperative clinical risk factors to predict intestinal resection in pediatric intussusception suspecting intestinal necrosis was developed and validated. Risk factors such as the duration of symptoms, CRP, WBCs, ascites detected under ultrasound were incorporated into the nomogram based on the training set, which was identified as highly predictive tool that could be applied directly by validation set.

Abdominal pain, vomiting, and bloody stool constitute the classic triad of intussusception, but are presented in less than 33% of children upon admission (16). In all the enrolled cases, only 34 (6.216%) cases manifested the typical triad, 106 (19.378%) patients had intermittent abdominal pain, 488 (89.214%) patients experienced vomiting, and 407 (74.406%) patients had bloody stools. However, out of 400 cases showing intermittent crying, 326 cases were younger than 12 months and unable to verbally complain about their pain, which may explain the low incidence of the typical triad. A study had shown that vomiting and fever lasting more than two days were important predictors of enterectomy (5). Vomiting before hospitalization was statistically associated with intestinal resection in our univariate analysis (OR = 2.966; 95% CI, 1.138–7.731; *p* = 0.026). Apart from the above typical symptoms, fever is also common in intussusception. In our cohort, 238 cases (43.51%) presented with fever, with a higher incidence in the intestinal resection group (74 out of 133 cases). Fever reflects a systemic response to intraperitoneal infection and inflammation. Furthermore, hyperpyrexia indicates a poor prognosis (17). Khorana et al. (18) drew a conclusion that a temperature above 37.8°C was one of the predictors of failed manual reduction. And a study on the retrospective analysis of 376 infants aged between 1 and 12 months from 19 states who suffered from intussusception showed that documented fever (temperature >38°C) on admission significantly increased the risk of bowel resection with the OR = 2.7 (adjusted for race and sex), 95% CI 1.2–6.0 (5). Another study conducted previously also identified fever (temperature >37.5°C) into risk factor for predicting intestinal necrosis (7). However, since the *p*-values in the multivariable analysis were higher than 0.05 and compromised the model's calibration, vomiting and fever were excluded from our model.

The study results were consistent with the findings of previous studies, indicating that a longer duration of illness was more common in patients with intestinal necrosis (7, 11, 12, 14, 19–21). Longer duration of symptoms before presentation is related to an increase in the loss of intestinal viability (18). However, differences were observed in the threshold values among various studies, ranging from 24 h to 75 h which may be attributed to recall bias. In our study, the mean duration of symptoms in non-intestinal resection group and intestinal resection group were found to be significantly different and intestinal resection was observed in most of cases with symptoms lasting more than 24 h. Therefore, further research is urgently needed to obtain a more accurate value.

As time prolongs, intestinal tract loses viability, evolving into ischemia and necrosis with massive amounts of toxins likely reaching the bloodstream and becoming measurable which attract attention for potential use in diagnosing intestinal necrosis at an early stage (22). Elevated CRP, WBCs, and other inflammatory factors are common. CRP level is a dynamic inflammation indicator well reflecting systemic inflammation elevating in response to inflammatory reactions, resistance to bacterial infections and tissue damage such as acute appendicitis (23), incarcerated groin hernia (10), intestinal obstruction (24), etc. As shown in our nomogram, CRP significantly contributed to intestinal necrosis, with higher CRP level greatly increasing the risk of intestinal necrosis and indicating the severity of the illness. Some predictive models also took CRP into consideration (25, 26) consistent with the current study. Total leukocyte count has traditionally been considered a hallmark of infection and inflammation (27). Our laboratory study suggested WBCs was an independent early predictor in diagnosing intestinal resection in pediatric intussusception suspecting intestinal necrosis (OR = 1.110; 95% CI, 1.048–1.176; *p* = 0.002) and significantly higher in intestinal resection than non-intestinal resection, which was probably related to the prolongation of intestinal damage with massive leukocyte releasing into the bloodstream. Coherent with the observations we made, Chen et al. (24) concluded WBCs increased more in severe intestinal obstruction than less severe cases. A study conducted on childhood intussusception after surgery also came to the same conclusion that CRP and WBCs levels were higher in patients who underwent bowel resection than those who underwent manual surgical reduction merely (25). Wu et al. (11) study showed that WBCs level was higher in bowel resection than non-bowel resection, although the difference was not statistically significant due to small sample size. Delgado-Miguel et al. (28) found neutrophils-to-lymphocytes (NLR) a useful indicator of surgery intervention (AUC = 0.925, sensibility: 73.2%, specificity: 94.5%) and was higher in surgery group than non-surgery group, while no significantly differences were found about neutrophils and lymphocytes in our study which may related with different inclusion criteria and basis for grouping. However, they came to similar conclusions about CRP as we did which indicating CRP may be a sensitive indictor.

Inflammation is widely regarded as a regulator of coagulation and fibrinolytic activity. As the terminal products of fibrin degradation, D-dimer increases *in vivo* during a hypercoagulable state and secondary hyperfibrinolysis, including disseminated intravascular coagulation (DIC), acute ischemic stroke, or acute venous thromboembolism, etc. (29, 30). The association between higher D-dimer level and intestinal ischemia has been reported previously (26, 29, 31). Our study likewise demonstrated that D-dimer had a significant positive correlation with intestinal resection (OR = 1.128; 95% CI, 1.005–1.267; *p* = 0.040) in univariable analysis, whereas no significant difference was observed in the multivariate analysis. Thus, we got it rid of our model.

Ultrasound is the primary imaging modality used to diagnose intussusception, with a sensitivity of 98%–100%, a specificity of 88%–100%, and a negative predictive value of 100% (2, 32, 33). Besides the essentiality of diagnosis, ultrasonography also provides important elements in the therapeutic choice of non-surgical or surgical reduction. Absence of vascular signs on color-Doppler and trapped fluid between involved bowels highlight the necessity of surgery (2). It was reported that the occurrence of fluid retention between the intussuscepted bowel segments strongly correlated with bowel ischemia, with a sensitivity 80%–100%, a specificity of 75%–92% (34, 35), while the presence of free intraperitoneal fluid did not essentially indicate the existence of complications such as peritonitis, and intestinal necrosis or perforation (2). Hosokawa et al. (36) also showed echogenic ascites was associated with surgical interventions in intestinal twist. And in our study, ascites detected by abdominal ultrasound is one of the independent predictors of intestinal necrosis in intussusception which may attribute to intestinal wall venous and lymphatic congestion resulting from intestinal wall compression after prolonged intussusception (3, 34).

However, our study had some limitations. First, it was a retrospective, observational study, so the accuracy of medical records could not be assessed. Second, the sample size was relatively small, and exclusively patients with air-enema failure were screened, which limited the generalizability and representativeness of the conclusions to some extent. Further long-term, prospective studies with large samples are urgently needed.

## 5 Conclusion

In conclusion, we developed a nomogram on the basis of clinical risk factors for intestinal resection in pediatric intussusception suspecting intestinal necrosis. This nomogram can be conveniently employed to facilitate preoperative decision-making for pediatric intussusception.

## Data Availability

The raw data supporting the conclusions of this article will be made available by the authors, without undue reservation.

## References

[1] Somme S To T Langer JC. Factors determining the need for operative reduction in children with intussusception: a population-based study. J Pediatr Surg. (2006) 41:1014–9. 10.1016/j.jpedsurg.2005.12.047 16677903

[2] Bartocci M Fabrizi G Valente I Manzoni C Speca S Bonomo L. Intussusception in childhood: role of sonography on diagnosis and treatment. J Ultrasound. (2015) 18:205–11. 10.1007/s40477-014-0110-9 26261462 PMC4529406

[3] Bogdanović M Blagojević M Kuzmanović J Ječmenica D Alempijević Đ. Fatal intussusception in infancy: forensic implications. Forensic Sci Med Pathol. (2019) 15:284–7. 10.1007/s12024-018-0039-y 30397871

[4] Huppertz H-I Soriano-Gabarró M Grimprel E Franco E Mezner Z Desselberger U Intussusception among young children in Europe. Pediatr Infect Dis J. (2006) 25:S22–9. 10.1097/01.inf.0000197713.32880.46 16397426

[5] Johnson B Gargiullo P Murphy TV Parashar UD Patel MM. Factors associated with bowel resection among infants with intussusception in the United States. Pediater Emerg Care. (2012) 28:529–32. 10.1097/PEC.0b013e3182587d12 22653458

[6] Ademuyiwa A Alakaloko F Elebute O Bode C Udenze I. Serum intestinal fatty-acid binding protein: predictor of bowel necrosis in pediatric intussusception. J Pediatr Surg. (2018) 53:335–8. 10.1016/j.jpedsurg.2017.11.028 29208308

[7] Huang HY Huang XZ Han YJ Zhu LB Huang KY Lin J Risk factors associated with intestinal necrosis in children with failed non-surgical reduction for intussusception. Pediatr Surg Int. (2017) 33:575–80. 10.1007/s00383-017-4060-0 28124113

[8] Iasonos A Schrag D Raj GV Panageas KS. How to build and interpret a nomogram for cancer prognosis. J Clin Oncol. (2008) 26:1364–70. 10.1200/jco.2007.12.9791 18323559

[9] Sagna A Camara S Ly S Fall I. Acute intestinal intussusception of the infant and the child: a 5-year study of 66 cases. Afr J Paediatr Surg. (2018) 15:138–41. 10.4103/ajps.AJPS_127_15 32769365 PMC7646681

[10] Yildirim M Dasiran F Angin YS Okan I. Lymphocyte-C-reactive protein ratio: a putative predictive factor for intestinal ischemia in strangulated abdominal wall hernias. Hernia. (2021) 25:733–9. 10.1007/s10029-020-02174-x 32222842

[11] Wu TH Huang GS Wu CT Lai JY Chen CC Hu MH. Clinical characteristics of pediatric intussusception and predictors of bowel resection in affected patients. Front Surg. (2022) 9:926089. 10.3389/fsurg.2022.926089 36111223 PMC9468224

[12] Wong CWY Jin S Chen J Tam PKH Wong KKY. Predictors for bowel resection and the presence of a pathological lead point for operated childhood intussusception: a multi-center study. J Pediatr Surg. (2016) 51:1998–2000. 10.1016/j.jpedsurg.2016.09.033 27666006

[13] Wang X Lu J Song Z Zhou Y Liu T Zhang D. From past to future: bibliometric analysis of global research productivity on nomogram (2000–2021). Front Public Health. (2022) 10:997713. 10.3389/fpubh.2022.997713 36203677 PMC9530946

[14] Zhuang Y Wang X Fan X Li F He G Luo M Developing a nomogram for predicting surgical intervention in pediatric intussusception after hydrostatic reduction. Front Pediatr. (2023) 11:1092548. 10.3389/fped.2023.1092548 37325352 PMC10264573

[15] Ting X Xufei D Jiangbin L Weijue X Zhibao L Guogang Y. Development and validation of a nomogram for predicting pathological intussusceptions in children prior to surgical intervention. Front Pediatr. (2022) 10:877358. 10.3389/fped.2022.877358 35923785 PMC9339649

[16] Simon NM Joseph J Philip RR Sukumaran TU Philip R. Intussusception: single center experience of 10 years. Indian Pediatr. (2019) 56:29–32. 10.1007/s13312-019-1462-1 30806357

[17] Stringer MD Pablot SM Brereton RJ. Paediatric intussusception. Brit J Surg. (1992) 79:867–76. 10.1002/bjs.1800790906 1422744

[18] Khorana J Singhavejsakul J Ukarapol N Laohapensang M Siriwongmongkol J Patumanond J. Prognostic indicators for failed nonsurgical reduction of intussusception. Ther Clin Risk Manag. (2016) 12:1231–7. 10.2147/tcrm.S109785 27563245 PMC4984823

[19] Nisar MU Sikander S Khan NA Javed N Chaudhary MA Saif MAA. Determinants of bowel resection in childhood intussusception. J Ayub Med Coll Abbottabad. (2020) 32:9–12. 32468746

[20] Liu ST Li YF Wu QY Ma X Bai YZ. Is enema reduction in pediatric intussusception with a history of over 48 h safe: a retrospective cohort study. Am J Emerg Med. (2023) 68:33–7. 10.1016/j.ajem.2023.02.027 36905884

[21] Wang Y Zhao R Xia L Cui Y-P Zhou Y Wu X-T. Predictive risk factors of intestinal necrosis in patients with mesenteric venous thrombosis: retrospective study from a single center. Can J Gastroenterol. (2019) 2019:8906803. 10.1155/2019/8906803 PMC6530214 31205904

[22] Matsumoto S Sekine K Funaoka H Yamazaki M Shimizu M Hayashida K Diagnostic performance of plasma biomarkers in patients with acute intestinal ischaemia. Brit J Surg. (2014) 101:232–8. 10.1002/bjs.9331 24402763

[23] Chen Y Wang ZY Xiao D Zeng HW Ma XP. Predicting the severity of acute appendicitis of young children (<3 years old): development and assessment of a new prediction nomogram. Front Pediatr. (2021) 9:763125. 10.3389/fped.2021.763125 34869120 PMC8637160

[24] Chen W Xiao J Yan J Liu R Yang J Xiao Y Analysis of the predictors of surgical treatment and intestinal necrosis in children with intestinal obstruction. J Pediatr Surg. (2020) 55:2766–71. 10.1016/j.jpedsurg.2020.07.017 32829882

[25] Chen B Cao J Yan C Zheng C Chen J Guo C. A promising new predictive factor for detecting bowel resection in childhood intussusception: the lymphocyte-C-reactive protein ratio. BMC Pediatr. (2021) 21:577. 10.1186/s12887-021-03068-2 34915876 PMC8675458

[26] Huang HY Lin XK Guo SK Bao XZ Lin ZX Li ZR Haemostatic indexes for predicting intestinal necrosis in children with intussusception. ANZ J Surg. (2021) 91:1485–90. 10.1111/ans.16854 33908173

[27] Tanrikulu Y Sen Tanrikulu C Sabuncuoglu MZ Temiz A Kokturk F Yalcin B. Diagnostic utility of the neutrophil-lymphocyte ratio in patients with acute mesenteric ischemia: a retrospective cohort study. Ulus Travma Acil Cerrahi Derg. (2016) 22:344–9. 10.5505/tjtes.2015.28235 27598606

[28] Delgado-Miguel C García A Delgado B Muñoz-Serrano AJ Miguel-Ferrero M Camps J Neutrophil-to-lymphocyte ratio as a predictor of the need for surgical treatment in children’s intussusception. Eur J Pediatr Surg. (2023) 33:422–7. 10.1055/a-1913-4280 35913089

[29] Yang K Wang W Zhang WH Chen XL Zhou J Chen XZ The combination of D-dimer and peritoneal irritation signs as a potential indicator to exclude the diagnosis of intestinal necrosis. Medicine (Baltimore). (2015) 94:e1564. 10.1097/md.0000000000001564 26448003 PMC4616729

[30] Favresse J Lippi G Roy P-M Chatelain B Jacqmin H Cate HT D-dimer: preanalytical, analytical, postanalytical variables, and clinical applications. Crit Rev Cl Lab Sci. (2018) 55:548–77. 10.1080/10408363.2018.1529734 30694079

[31] Srettabunjong S. Fatal acute hemorrhagic bowel infarction caused by mesenteric venous thrombosis. J Forensic Sci. (2018) 63:305–8. 10.1111/1556-4029.13525 28425094

[32] Sacks RS Anconina R Farkas E Zolotnik-Krupenich D Kravarusic D Tsodikov V Sedated ultrasound guided saline reduction (SUR) of ileocolic intussusception: 20 year experience. J Pediatr Surg. (2020) 55:2009–14. 10.1016/j.jpedsurg.2020.05.049 32713713

[33] Hryhorczuk AL Strouse PJ. Validation of US as a first-line diagnostic test for assessment of pediatric ileocolic intussusception. Pediatr Radiol. (2009) 39:1075–9. 10.1007/s00247-009-1353-z 19657636

[34] del-Pozo G Albillos JC Tejedor D Calero R Rasero M de-la-Calle U Intussusception in children: current concepts in diagnosis and enema reduction. Radiographics. (1999) 19:299–319. 10.1148/radiographics.19.2.g99mr14299 10194781

[35] Normahayu I Pramusinta W Andarini S Yueniwati Y. The presence of trapped fluid on ultrasound as high predictive value for intestinal necrosis in pediatric intussusception. GSC Adv Res Rev. (2021) 08(01):053–9. 10.30574/gscarr.2021.8.1.0142

[36] Hosokawa T Hosokawa M Tanami Y Sato Y Ishimaru T Tanaka Y Use of ultrasound findings to predict bowel ischemic changes in pediatric patients with intestinal volvulus. J Ultras Med. (2020) 39:683–92. 10.1002/jum.15145 31642550

